# Fixed point results of weakly contraction mappings in partially ordered *b*-metric spaces

**DOI:** 10.1186/s13104-022-05914-7

**Published:** 2022-02-09

**Authors:** K. Kalyani, N. Seshagiri Rao

**Affiliations:** 1 Department of Mathematics, Vignan’s Foundation for Science, Technology & Research, Vadlamudi, Andhra Pradesh 522213 India; 2 Department of Applied Mathematics, School of Applied Natural Sciences, Adama Science and Technology University, Post Box No.1888, Adama, Ethiopia

**Keywords:** $({\hat{\phi }}, {\hat{\psi }})$-Weak contractions, Fixed point, Coincidence point, Compatible mappings, Coupled coincidence point, Coupled common fixed points, Primary: 54H25, Secondary: 47H10

## Abstract

**Objectives:**

We explored the results of fixed point, coincidence point and coupled coincidence point for the mappings in an ordered metric spaces. Our results generalized and extended the well-known results in the literature. Some numerical examples are provided for justifying the results obtained.

**Result:**

Some fixed point results are found for a self mapping in a partially ordered *b*-metric space which satisfies a generalized week contraction condition. Furthermore, these results are extended for two self mappings for obtaining coincidence point, coupled coincidence point and coupled common fixed point in the same context. A few examples are presented to support the findings.

## Introduction

A *b*-metric space also referred to as a metric type space by some researchers is one of the most influential generalizations of ordinary metric space. It has a wide range of uses in mathematical research and scientific applications. It was first established by Bakhtin in [7] and eventually expanded upon by Czerwik in [10]. Later, Aghajani et al. [1], Allahyari et al. [5] investigated some fixed point results of generalized contractive mappings in partially ordered *b*-metric space and then applied their results to quadratic integral equations. Common fixed point results for generalized weak contractions in the same context was studied by Aghajani et al. [2]. Also, the results on common fixed point for two self mappings under an implicit relation was explored by Akkouchi [3]. Some remarks on fixed point results in *b*-metric space were discussed by Aleksić et al. [4]. Common fixed point for weak $\varphi$-contractions on *b*-metric spaces was examined by Aydi et al. [6]. Recently, some results on fixed point, coincidence point, coupled coincidence point for the self mappings satisfying generalized weak contractions have been discussed by Belay et al. [8], Seshagiri Rao et al. [20, 24, 25] in partially ordered *b*-metric space with necessary topological properties.

In this paper, we introduced the following generalized weak contraction condition which involve the altering distance functions ${\hat{\phi }} \in {\hat{\Phi }}$, ${\hat{\psi }} \in {\hat{\Psi }}$ defined below to acquire a fixed point of a mapping ${\mathscr {L}}{:}{\mathfrak {P}} \rightarrow {\mathfrak {P}}$ in a partially ordered *b*-metric space

1 $$ {\hat{\phi }}({\mathcalligra{s}}\eth ({\mathscr {L}}\zeta ,{\mathscr {L}}\varpi ))\le {\hat{\phi }}({\mathcal {C}}(\zeta ,\varpi ))-{\hat{\psi }}({\mathcal {D}}(\zeta ,\varpi )),$$

for any $\zeta ,\varpi \in {\mathfrak {P}}$ with $\zeta \preceq \varpi$, ${\mathcalligra {s}}>1$ and, where

$$ {\mathcal {C}}(\zeta ,\varpi )=\max \left\{\frac{\eth (\varpi ,{\mathscr {L}}\varpi ) \left[ 1+\eth (\zeta ,{\mathscr {L}}\zeta )\right] }{1+\eth (\zeta ,\varpi )},\frac{\eth (\zeta ,{\mathscr {L}}\zeta )~\eth (\varpi ,{\mathscr {L}}\varpi )}{1+\eth (\zeta ,\varpi )}, \eth (\zeta ,{\mathscr {L}}\zeta ),\eth (\varpi ,{\mathscr {L}}\varpi ), \eth (\zeta ,\varpi )\right\} $$

and

$$ {\mathcal {D}}(\zeta ,\varpi )=\max \left\{\frac{\eth (\varpi ,{\mathscr {L}}\varpi ) \left[ 1+\eth (\zeta ,{\mathscr {L}}\zeta )\right] }{1+\eth (\zeta ,\varpi )}, \eth (\zeta ,\varpi )\right\}.$$

Also generalized this result by involving two self mappings in the above generalized contraction condition to obtain a coincidence point, coupled coincidence point and common fixed point for the mappings in the same context. Our results are generalized and extended the results of Belay et al. [8], Bhaskar et al. [9], Harjani et al. [12] and Jachymski [14] and Seshagiri Rao et al. [20, 24, 25]. The authors may refer the papers of Aghajani et al. [1], Dorić et al. [11], Huaping Huang et al. [13], Roshan et al. [18, 19] and Seshagiri Rao et al. [21–23] for the basic definitions and necessary results which we used in the present study.

In the whole paper, we use the following nations for the altering distance functions: ${\hat{\Phi}}=\{{\hat{\phi }}/ {\hat{\phi}}\;\text {is}\text{ continuous},\;\text{non-decrasing}\;\text{self}\;\text{mapping}\;\text{on}\; [0, +\infty) \;\text {with}\; {\hat{\phi }}(\varepsilon )=0\;\text {iff}\;\varepsilon =0,\; \text {for}\; \varepsilon \in [0, +\infty ) \}$ and ${\hat{\Psi }}=\{{\hat{\psi }}/ {\hat{\psi }}\;\text {is}\;\text {lower}\;\text {semi-continuous}\;\text {self}\;\text {mapping}\text {on}\; [0, +\infty )\; \text {such}\; \text {that}\;{\hat{\psi }}(\varepsilon )=0\;\text {if}\;\text {and}\;\text {only}\;\text {if}\;\varepsilon =0, \text {where}\;\varepsilon \in [0, +\infty) \}$.

## Main results

We start this section with the following fixed point result in a complete partially ordered *b*-metric space.

### **Theorem 2.1**

*Suppose* $({\mathfrak {P}},\eth ,\preceq )$ *be a complete partially ordered b-metric space with* ${\mathcalligra {s}} > 1.$ *Assume that a continuous self mapping* ${\mathscr {L}}$ *on* ${\mathfrak {P}}$ *is non-decreasing with respect to* $\preceq$ *and satisfies the condition (*1*). If for some* $\zeta _0 \in {\mathfrak {P}}$ *such that* $\zeta _0 \preceq {\mathscr {L}}\zeta _0,$ *then* ${\mathscr {L}}$ *has a fixed point in* ${\mathfrak {P}}.$

### *Proof*

The proof is trivial for ${\mathscr {L}}\zeta _0=\zeta _0$, for some $\zeta _0 \in {\mathfrak {P}}$. Suppose not then $\zeta _0 \prec {\mathscr {L}}\zeta _0$. Now define a sequence $\{\zeta _n\} \subset {\mathfrak {P}}$ by $\zeta _{n+1}={\mathscr {L}}\zeta _n$, for $n\ge 0$. Since ${\mathscr {L}}$ is non-decreasing then

2 $$\begin{aligned} \zeta _0 \prec {\mathscr {L}}\zeta _0=\zeta _1\preceq \cdots\preceq \zeta _n \preceq {\mathscr {L}}\zeta _n=\zeta _{n+1}\preceq \cdots. \end{aligned}$$

If for some $n_0\in {\mathbb {N}}$, $\zeta _{n_0}=\zeta _{n_0+1}$, then from (2), ${\mathscr {L}}$ has a fixed point $\zeta _{n_0}$. Assume that $\zeta _n \ne \zeta _{n+1}$ for all $n \ge 1$. Since $\zeta _n>\zeta _{n-1}$ for all $n \ge 1$, then from (1), we have

3 $$\begin{aligned} {\hat{\phi }}(\eth (\zeta _n,\zeta _{n+1})) &= {\hat{\phi }}(\eth ({\mathscr {L}}\zeta _{n-1},{\mathscr {L}}\zeta _n)) \le {\hat{\phi }}({\mathcalligra {s}}\eth ({\mathscr {L}}\zeta _{n-1},{\mathscr {L}}\zeta _n)) \\ & \le {\hat{\phi }}({\mathcal {C}}(\zeta _{n-1},\zeta _n))-{\hat{\psi }}({\mathcal {D}}(\zeta _{n-1},\zeta _n)). \end{aligned} $$

Thus from (3), we have

4 $$ \eth (\zeta _n,\zeta _{n+1})= \eth ({\mathscr {L}}\zeta _{n-1},{\mathscr {L}}\zeta _n)\le \frac{1}{{\mathcalligra {s}}} {\mathcal {C}}(\zeta _{n-1},\zeta _n), $$

where

$$ \begin{aligned} {\mathcal {C}}(\zeta _{n-1},\zeta _n)&=\max \left\{\frac{\eth (\zeta _n,{\mathscr {L}}\zeta _n) \left[ 1+\eth (\zeta _{n-1},{\mathscr {L}}\zeta _{n-1})\right] }{1+\eth (\zeta _{n-1},\zeta _n)}, \frac{\eth (\zeta _{n-1},{\mathscr {L}}\zeta _{n-1})~\eth (\zeta _n,{\mathscr {L}}\zeta _n)}{1+\eth (\zeta _{n-1},\zeta _n)}, \eth (\zeta _{n-1},{\mathscr {L}}\zeta _{n-1}),\eth (\zeta _n,{\mathscr {L}}\zeta _n), \eth (\zeta _{n-1},\zeta _n)\right\} \\ & = \max \left\{\eth (\zeta _n,\zeta _{n+1}), \frac{\eth (\zeta _{n-1},\zeta _n)\;\eth (\zeta _n,\zeta _{n+1})}{1+\eth (\zeta _{n-1},\zeta _n)}, \eth (\zeta _{n-1},\zeta _n)\right\} \\ & \le \max \{\eth (\zeta _n,\zeta _{n+1}),\eth (\zeta _{n-1},\zeta _n)\}. \end{aligned}$$

If $\max \{\eth (\zeta _n,\zeta _{n+1}),\eth (\zeta _{n-1}, \zeta _n)\}= \eth (\zeta _n,\zeta _{n+1})$ for some $n \ge 1$, then from (4), we have

$$ \eth (\zeta _n,\zeta _{n+1})\le \frac{1}{{\mathcalligra {s}}} \eth (\zeta _n,\zeta _{n+1}), $$

this is a contradiction. Hence, $\max \{\eth (\zeta _n,\zeta _{n+1}),\eth (\zeta _{n-1},\zeta _n)\}= \eth (\zeta _{n-1},\zeta _n)$ for all $n \ge 1$. Thus from (4) we have

5 $$ \eth (\zeta _n,\zeta _{n+1})\le \frac{1}{{\mathcalligra {s}}} \eth (\zeta _{n-1},\zeta _n). $$

Since $\frac{1}{{\mathcalligra {s}}}\in (0,1)$ then $\{\zeta _n\}$ is a Cauchy sequence from [4, 6]. Also, the completeness of ${\mathfrak {P}}$ implies that $\zeta _n \rightarrow \varepsilon$ for some $\varepsilon \in {\mathfrak {P}}$ .

Furthermore the continuity of ${\mathscr {L}}$ implies that,

$$ {\mathscr {L}}\varepsilon ={\mathscr {L}}(\lim \limits _{n\rightarrow +\infty }\zeta _n)=\lim \limits _{n\rightarrow +\infty }{\mathscr {L}}\zeta _n=\lim \limits _{n\rightarrow +\infty }\zeta _{n+1}=\varepsilon , $$

which shows that ${\mathscr {L}}$ has a fixed point $\varepsilon \in {\mathfrak {P}}$. $\square$

We have the following result in which the mapping ${\mathscr {L}}$ is not continuous, still is valid to have a fixed point.

### **Theorem 2.2**

*According to Theorem* 2.1*, a non-continuous self mapping* ${\mathscr {L}}$ *has a fixed point if* ${\mathfrak {P}}$ *meets the condition (*6*):*

6 $$ \begin{aligned}& \text {a}\;\text {non-decreasing}\;\text {sequence}\; \{\zeta _n\} \subseteq {\mathfrak {P}}\; \text {such}\; \text {that}\; \zeta _n\rightarrow \varepsilon \in {\mathfrak {P}}\; \text {then}\; \zeta _n \preceq \varepsilon \; \text {for}\; n \in {\mathbb {N}},\\ & \text {that}\;\text {is},\; \varepsilon =\sup \zeta _n. \end{aligned} $$

### *Proof*

As from Theorem 2.1, a non-decreasing Cauchy sequence $\{\zeta _n\} \subseteq {\mathfrak {P}}$ exists such that $\zeta _n \rightarrow \varepsilon \in {\mathfrak {P}}$. Hence from condition (6), $\zeta _n \preceq \varepsilon$ for all *n*, i.e., $\varepsilon =\sup \zeta _n$.

Next to show that $\varepsilon$ is a fixed point of ${\mathscr {L}}$ in ${\mathfrak {P}}$. Suppose that ${\mathscr {L}}\varepsilon \ne \varepsilon$. Let

$${\mathcal {C}}(\zeta _n,\varepsilon )=\max \left\{\frac{\eth (\varepsilon ,{\mathscr {L}}\varepsilon ) \left[ 1+\eth (\zeta _n,{\mathscr {L}}\zeta _n)\right] }{1+\eth (\zeta _n,\varepsilon )}, \frac{\eth (\zeta _n,{\mathscr {L}}\zeta _n)~\eth (\varepsilon ,{\mathscr {L}}\varepsilon ) }{1+\eth (\zeta _n,\varepsilon )}, \eth (\zeta _n,{\mathscr {L}}\zeta _n),\eth (\varepsilon ,{\mathscr {L}}\varepsilon ), \eth (\zeta _n,\varepsilon )\right\}$$

and

$$ {\mathcal {D}}(\zeta _n,\varepsilon )=\max \left\{\frac{\eth (\varepsilon ,{\mathscr {L}}\varepsilon ) \left[ 1+\eth (\zeta _n,{\mathscr {L}}\zeta _n)\right] }{1+\eth (\zeta _n,\varepsilon )}, \eth (\zeta _n,\varepsilon )\right\}. $$

As $n\rightarrow +\infty$ and the fact that $\lim \limits _{n\rightarrow +\infty }\zeta _n=\varepsilon$, we obtain that

7 $$ \lim \limits _{n \rightarrow +\infty }{\mathcal {C}}(\zeta _n, \varepsilon )= \max \{\eth (\varepsilon ,{\mathscr {L}}\varepsilon ),0\}=\eth (\varepsilon ,{\mathscr {L}}\varepsilon ), $$

and

8 $$ \lim \limits _{n \rightarrow +\infty }{\mathcal {D}}(\zeta _n, \varepsilon )= \max \{\eth (\varepsilon ,{\mathscr {L}}\varepsilon ),0\}=\eth (\varepsilon ,{\mathscr {L}}\varepsilon ). $$

Since $\zeta _n \preceq \varepsilon$ for any *n*, then (1) becomes

9 $$ {\hat{\phi }}(\eth (\zeta _{n+1}, {\mathscr {L}}\varepsilon ))={\hat{\phi }} (\eth ({\mathscr {L}}\zeta _n, {\mathscr {L}}\varepsilon ))\le {\hat{\phi }}({\mathcalligra {s}} \eth ({\mathscr {L}}\zeta _n, {\mathscr {L}}\varepsilon )\le {\hat{\phi }}({\mathcal {C}}(\zeta _n, \varepsilon ))-{\hat{\psi }}({\mathcal {D}}(\zeta _n, \varepsilon )). $$

Taking $n \rightarrow +\infty$ in (9) and from Eqs. (7) and (8), we get

$$ {\hat{\phi }}(\eth (\varepsilon,{\mathscr {L}}\varepsilon )) \le {\hat{\phi }}(\eth (\varepsilon ,{\mathscr {L}}\varepsilon ))-{\hat{\psi }}(\eth (\varepsilon, {\mathscr {L}}\varepsilon ))< {\hat{\phi }}(\eth (\varepsilon ,{\mathscr {L}}\varepsilon )),$$

which is a contradiction. Hence, ${\mathscr {L}}\varepsilon =\varepsilon$, i.e., ${\mathscr {L}}$ has a fixed point $\varepsilon$ in ${\mathfrak {P}}$. $\square$

### **Theorem 2.3**

*If every two elements of* ${\mathfrak {P}}$ *are comparable then* ${\mathscr {L}}$ *has a unique fixed point in Theorems* 2.1*and* 2.2*.*

### *Proof*

Let $\zeta ^*\ne \varpi ^*$ be two fixed points of ${\mathscr {L}}$ in ${\mathfrak {P}}$, then from (1), we have

$${\hat{\phi }}(\eth ({\mathscr {L}}\zeta ^*, {\mathscr {L}}\varpi ^*)) \le {\hat{\phi }}({\mathcalligra {s}}\eth ({\mathscr {L}}\zeta ^*, {\mathscr {L}}\varpi ^*)) \le {\hat{\phi }}({\mathcal {C}}(\zeta ^*, \varpi ^*))-{\hat{\psi }}({\mathcal {D}}(\zeta ^*, \varpi ^*)). $$

As a result, we get

10 $$\eth (\zeta ^*, \varpi ^*)= \eth ({\mathscr {L}}\zeta ^*, {\mathscr {L}}\varpi ^*) \le \frac{1}{{\mathcalligra {s}}} {\mathcal {C}}(\zeta ^*, \varpi ^*), $$

where

$$\begin{aligned}  {\mathcal {C}}(\zeta ^*,\varpi ^*)& =\max \left\{\frac{\eth (\varpi ^*,{\mathscr {L}}\varpi ^*) \left[ 1+\eth (\zeta ^*,{\mathscr {L}}\zeta ^*)\right] }{1+\eth (\zeta ^*,\varpi ^*)}, \frac{\eth (\zeta ^*,{\mathscr {L}}\zeta ^*)~\eth (\varpi ^*,{\mathscr {L}}\varpi ^*)}{1+\eth (\zeta ^*,\varpi ^*)}, \eth (\zeta ^*,{\mathscr {L}}\zeta ^*),\eth (\varpi ^*,{\mathscr {L}}\varpi ^*), \eth (\zeta ^*,\varpi ^*)\right\} \\ & =\max \left\{\frac{\eth (\varpi ^*,\varpi ^*) \left[ 1+\eth (\zeta ^*,\zeta ^*)\right] }{1+\eth (\zeta ^*,\varpi ^*)},\frac{\eth (\zeta ^*,\zeta ^*)~\eth (\varpi ^*,\varpi ^*)}{1+\eth (\zeta ^*,\varpi ^*)}, \eth (\zeta ^*,\zeta ^*),\eth (\varpi ^*,\varpi ^*), \eth (\zeta ^*,\varpi ^*)\right\} \\ & = \max \{0, \eth (\zeta ^*,\varpi ^*) \} \\ & =\eth (\zeta ^*,\varpi ^*).  \end{aligned}$$

Therefore from (10), we have

$$ \eth (\zeta ^*, \varpi ^*) \le \frac{1}{{\mathcalligra {s}}} \eth (\zeta ^*, \varpi ^*)<\eth (\zeta ^*, \varpi ^*), $$

which leads contradiction to $\zeta ^*\ne \varpi ^*$. Thus, $\zeta ^*= \varpi ^*$. $\square$

We have the following consequences from Theorems 2.1, 2.2 and 2.3.

### **Corollary 2.4**

*Instead* ${\mathcal {D}}(\zeta ,\varpi )$ *by* ${\mathcal {C}}(\zeta ,\varpi )$ *in condition (*1*), we have the same conclusions as from Theorems* 2.1*,* 2.2*and* 2.3*.*

### **Corollary 2.5**

*Taking* ${\hat{\phi }}(m)=m$ *and* ${\hat{\psi }}(m)=(1-k)m$ *in Corollary* 2.4*, then the contraction condition becomes*

$$ \eth ({\mathscr {L}}\zeta ,{\mathscr {L}}\varpi )\le {\frac{k}{\mathcalligra{s}}}\max \left\{\frac{\eth (\varpi ,{\mathscr {L}}\varpi ) \left[ 1+\eth (\zeta ,{\mathscr {L}}\zeta )\right] }{1+\eth (\zeta ,\varpi )},\frac{\eth (\zeta ,{\mathscr {L}}\zeta )\;\eth (\varpi ,{\mathscr {L}}\varpi )}{1+\eth (\zeta ,\varpi )}, \eth (\zeta ,{\mathscr {L}}\zeta ),\eth (\varpi ,{\mathscr {L}}\varpi ), \eth (\zeta ,\varpi )\right\}. $$

*Then one can arrive at the same conclusions as in Theorems* 2.1*,* 2.2*and* 2.3*.*

A self mapping ${\mathscr {L}}$ on ${\mathfrak {P}}$ with respect to ${\mathcalligra {f}}:{\mathfrak {P}} \rightarrow {\mathfrak {P}}$ is a generalized contraction mapping, if it satisfies the following condition for all $\zeta ,\varpi \in {\mathfrak {P}}$ with ${\mathcalligra {f}}\zeta \preceq {\mathcalligra {f}}\varpi$, ${\hat{\phi }}\in {\hat{\Phi }}$ and ${\hat{\psi }}\in {\hat{\Psi }}$:

11 $$ {\hat{\phi }}({\mathcalligra {s}}\eth ({\mathscr {L}}\zeta ,{\mathscr {L}}\varpi ))\le {\hat{\phi }}({\mathcal {C}}_{\mathcalligra {f}}(\zeta ,\varpi ))-{\hat{\psi }}({\mathcal {D}}_{\mathcalligra {f}}(\zeta ,\varpi )), $$

where

12 $$ {\mathcal {C}}_{\mathcalligra {f}}(\zeta ,\varpi )=\max \left\{\frac{\eth ({\mathcalligra {f}}\varpi ,{\mathscr {L}}\varpi ) \left[ 1+\eth ({\mathcalligra {f}}\zeta ,{\mathscr {L}}\zeta )\right] }{1+\eth ({\mathcalligra {f}}\zeta ,{\mathcalligra {f}}\varpi )}, \frac{\eth ({\mathcalligra {f}}\zeta ,{\mathscr {L}}\zeta )\;\eth ({\mathcalligra {f}}\varpi ,{\mathscr {L}}\varpi )}{1+\eth ({\mathcalligra {f}}\zeta ,{\mathcalligra {f}}\varpi )},\eth ({\mathcalligra {f}}\zeta ,{\mathscr {L}}\zeta ),\eth ({\mathcalligra {f}}\varpi ,{\mathscr {L}}\varpi ), \eth ({\mathcalligra {f}}\zeta ,{\mathcalligra {f}}\varpi )\right\}, $$

and

13 $$ {\mathcal {D}}_{\mathcalligra {f}}(\zeta ,\varpi )=\max \{\frac{\eth ({\mathcalligra {f}}\varpi ,{\mathscr {L}}\varpi ) \left[ 1+\eth ({\mathcalligra {f}}\zeta ,{\mathscr {L}}\zeta )\right] }{1+\eth ({\mathcalligra {f}}\zeta ,{\mathcalligra {f}}\varpi )}, \eth ({\mathcalligra {f}}\zeta ,{\mathcalligra {f}}\varpi )\}. $$

Now, we have the following result.

### **Theorem 2.6**

*The two continuous self-mappings* ${\mathscr {L}},{\mathcalligra {f}}$ *on* ${\mathfrak {P}}$ *have a coincidence point, if they satisfy the following conditions:*

- ${\mathscr {L}}$ *is a monotone* ${\mathcalligra {f}}$*-non-decreasing,*
- ${\mathscr {L}}{\mathfrak {P}} \subseteq {\mathcalligra {f}}{\mathfrak {P}}$ *and a pair* $({\mathscr {L}},{\mathcalligra {f}})$ *are compatible,*
- ${\mathcalligra {f}}\zeta _0 \preceq {\mathscr {L}}\zeta _0$ *for some* $\zeta _0 \in {\mathfrak {P}}$ *and*
- *satisfies the condition (*11*) in a complete partially ordered b-metric space* $({\mathfrak {P}},\eth ,\preceq ).$

### *Proof*

From Theorem 2.2 of [5], we have the sequences $\{\zeta _n\}, \{\varpi _n\} \subseteq {\mathfrak {P}}$ with

14 $$\varpi _n={\mathscr {L}}\zeta _n={\mathcalligra {f}}\zeta _{n+1} \;\text {for}\;\text {all}\;n\ge 0, $$

for which

15 $$ {\mathcalligra {f}}\zeta _0 \preceq {\mathcalligra {f}}\zeta _1 \preceq \cdots \preceq {\mathcalligra {f}}\zeta _n \preceq {\mathcalligra {f}}\zeta _{n+1} \preceq\cdots. $$

Now from [5], we have to show that

16 $$ \eth (\varpi _n,\varpi _{n+1})\le \lambda \eth (\varpi _{n-1},\varpi _n),$$

for all $n \ge 1$ and where $\lambda \in [0, \frac{1}{{\mathcalligra {s}}})$.

From Eqs. (11), (14) and (15), we have

17 $$\begin{aligned} \begin{aligned} {\hat{\phi }}({\mathcalligra {s}}\eth (\varpi _n,\varpi _{n+1}))&={\hat{\phi }}({\mathcalligra {s}}\eth ({\mathscr {L}}\zeta _n,{\mathscr {L}}\zeta _{n+1})) \\ {}&\le {\hat{\phi }}({\mathcal {C}}_{\mathcalligra {f}}(\zeta _n,\zeta _{n+1}))-{\hat{\psi }}({\mathcal {D}}_{\mathcalligra {f}}(\zeta _n,\zeta _{n+1})), \end{aligned} \end{aligned}$$

where

$$\begin{aligned} {\mathcal {C}}_{\mathcalligra {f}}(\zeta _n,\zeta _{n+1})& =\max \left\{\frac{\eth ({\mathcalligra {f}}\zeta _{n+1},{\mathscr {L}}\zeta _{n+1}) \left[ 1+\eth ({\mathcalligra {f}}\zeta _n,{\mathscr {L}}\zeta _n)\right] }{1+\eth ({\mathcalligra {f}}\zeta _n,{\mathcalligra {f}}\zeta _{n+1})}, \frac{\eth ({\mathcalligra {f}}\zeta _n,{\mathscr {L}}\zeta _n)~\eth ({\mathcalligra {f}}\zeta _{n+1},{\mathscr {L}}\zeta _{n+1})}{1+\eth ({\mathcalligra {f}}\zeta _n,{\mathcalligra {f}}\zeta _{n+1})}, \eth ({\mathcalligra {f}}\zeta _n,{\mathscr {L}}\zeta _n),\eth ({\mathcalligra {f}}\zeta _{n+1},{\mathscr {L}}\zeta _{n+1}), \eth ({\mathcalligra {f}}\zeta _n,{\mathcalligra {f}}\zeta _{n+1})\right\} \\ & =\max \left\{\frac{\eth (\varpi _n,\varpi _{n+1}) \left[ 1+\eth (\varpi _{n-1},\varpi _n)\right] }{1+\eth (\varpi _{n-1},\varpi _n)}, \frac{\eth (\varpi _{n-1},\varpi _n)\;\eth (\varpi _n,\varpi _{n+1})}{1+\eth (\varpi _{n-1},\varpi _n)},\eth (\varpi _{n-1},\varpi _n),\eth (\varpi _n,\varpi _{n+1}), \eth (\varpi _{n-1},\varpi _n)\right\} \\ & \le \max \{\eth (\varpi _{n-1},\varpi _n),\eth (\varpi _n,\varpi _{n+1})\} \end{aligned} $$

and

$$\begin{aligned} {\mathcal {D}}_{\mathcalligra {f}}(\zeta _n,\zeta _{n+1})&=\max \left\{\frac{\eth ({\mathcalligra {f}}\zeta _{n+1},{\mathscr {L}}\zeta _{n+1}) \left[ 1+\eth ({\mathcalligra {f}}\zeta _n,{\mathscr {L}}\zeta _n)\right] }{1+\eth ({\mathcalligra {f}}\zeta _n,{\mathcalligra {f}}\zeta _{n+1})},\eth ({\mathcalligra {f}}\zeta _n,{\mathcalligra {f}}\zeta _{n+1})\right\} \\ &=\max \left\{\frac{\eth (\varpi _n,\varpi _{n+1}) \left[ 1+\eth (\varpi _{n-1},\varpi _n)\right] }{1+\eth (\varpi _{n-1},\varpi _n)},\eth (\varpi _{n-1},\varpi _n)\right\} \\ &=\max \{\eth (\varpi _{n-1},\varpi _n),\eth (\varpi _n,\varpi _{n+1})\}. \end{aligned} $$

From Eq. (17), we have

18 $$ {\hat{\phi }}({\mathcalligra {s}}\eth (\varpi _n,\varpi _{n+1}))\le {\hat{\phi }}(\max \{\eth (\varpi _{n-1},\varpi _n),\eth (\varpi _n,\varpi _{n+1})\})-{\hat{\psi }}(\max \{\eth (\varpi _{n-1},\varpi _n),\eth (\varpi _n,\varpi _{n+1})\}). $$

If $0<\eth (\varpi _{n-1},\varpi _n)\le \eth (\varpi _n,\varpi _{n+1})$ for some *n*, then Eq. (18) follows that

$$ {\hat{\phi }}({\mathcalligra {s}}\eth (\varpi _n,\varpi _{n+1}))\le {\hat{\phi }}(\eth (\varpi _n,\varpi _{n+1}))-{\hat{\psi }}(\eth (\varpi _n,\varpi _{n+1}))<{\hat{\phi }}(\eth (\varpi _n,\varpi _{n+1})), $$

or equivalently

$$ {\mathcalligra {s}}\eth (\varpi _n,\varpi _{n+1})\le \eth (\varpi _n,\varpi _{n+1}),$$

a contradiction. Therefore, from Eq. (18) we have

19 $$ {\mathcalligra {s}}\eth (\varpi _n,\varpi _{n+1})\le \eth (\varpi _{n-1},\varpi _n). $$

Hence, $\lambda \in [0,\frac{1}{{\mathcalligra {s}}})$ from (16). According to Lemma 3.1 of [15] and from Eq. (16), we have

$$ \lim \limits _{n \rightarrow +\infty }{\mathscr {L}}\zeta _{n}=\lim \limits _{n \rightarrow +\infty }{\mathcalligra {f}}\zeta _{n+1}=\mu ,~\text {for}~\mu \in {\mathfrak {P}}.$$

From condition (b), we have

20 $$ \lim \limits _{n \rightarrow +\infty }\eth ({\mathcalligra {f}}({\mathscr {L}}\zeta _n), {\mathscr {L}}({\mathcalligra {f}}\zeta _n))=0, $$

and the continuity of ${\mathscr {L}}$ and ${\mathcalligra {f}}$ we have,

21 $$\lim \limits _{n \rightarrow +\infty }{\mathcalligra {f}}({\mathscr {L}}\zeta _n)={\mathcalligra {f}}\mu ,\quad\lim \limits _{n \rightarrow +\infty } {\mathscr {L}}({\mathcalligra {f}}\zeta _n)={\mathscr {L}}\mu. $$

Furthermore,

22 $$ \frac{1}{{\mathcalligra {s}}}\eth ({\mathscr {L}}\mu ,{\mathcalligra {f}}\mu )\le \eth ({\mathscr {L}}\mu ,{\mathscr {L}}({\mathcalligra {f}}\zeta _n))+{\mathcalligra {s}} \eth ({\mathscr {L}}({\mathcalligra {f}}\zeta _n), {\mathcalligra {f}}({\mathscr {L}}\zeta _n))+{\mathcalligra {s}}\eth ({\mathcalligra {f}}({\mathscr {L}}\zeta _n), {\mathcalligra {f}}\mu ). $$

Thus, $\eth ({\mathscr {L}}v,{\mathcalligra {f}}v)=0$ as $n \rightarrow +\infty$ in (22) and hence the result.

$$\square $$

We have the following result without the continuity property of ${\mathcalligra {f}}$ and ${\mathscr {L}}$ in Theorem 2.6.

### **Theorem 2.7**

*If* ${\mathfrak {P}}$ *has the property in Theorem* 2.6*that*

$$\begin{aligned} &\text { a sequence}~ \{{\mathcalligra {f}}\zeta _n\}\subset {\mathfrak {P}}~\text {is a non-decreasing such that}~ \lim \limits _{n \rightarrow +\infty } {\mathcalligra {f}}\zeta _n={\mathcalligra {f}}\zeta \in {\mathcalligra {f}}{\mathfrak {P}},\text {and}~ \\ & {\mathcalligra {f}}{\mathfrak {P}}\subseteq {\mathfrak {P}}\;\text {is}\;\text {closed}\;\text {and}\; {\mathcalligra {f}}\zeta _n \preceq {\mathcalligra {f}}\zeta , {\mathcalligra {f}}\zeta \preceq {\mathcalligra {f}}({\mathcalligra {f}}\zeta )\;\text {for}\;n\;\text {and}\;{\mathcalligra {f}}\zeta _0 \preceq {\mathscr {L}}\zeta _0 \;\text {for}\;\text {some}\;\zeta _0 \in {\mathfrak {P}}, \end{aligned}$$

*then the weakly compatible mappings* ${\mathscr {L}},{\mathcalligra {f}}$ *have a coincidence point. Besides that, when* ${\mathscr {L}}$ *and* ${\mathcalligra {f}}$ *commute at their coincidence points, then* ${\mathscr {L}},{\mathcalligra {f}}$ *have a common fixed point in* ${\mathfrak {P}}.$

### *Proof*

As from Theorem 2.6, $\{\varpi _n\}=\{{\mathscr {L}}\zeta _n\}=\{{\mathcalligra {f}}\zeta _{n+1}\}$ is a Cauchy sequence. Since ${\mathcalligra {f}}{\mathfrak {P}}$ is closed then

$$ \lim \limits _{n \rightarrow +\infty }{\mathscr {L}}\zeta _{n}=\lim \limits _{n \rightarrow +\infty }{\mathcalligra {f}}\zeta _{n+1}={\mathcalligra {f}}\mu \; \text {for}\; \mu \in {\mathfrak {P}}. $$

Thus, ${\mathcalligra {f}}\zeta _n\preceq {\mathcalligra {f}}\mu$ for all *n*. Next to show that ${\mathscr {L}},{\mathcalligra {f}}$ have a coincidence point $\mu$. From (11), we have

23 $$ {\hat{\phi }}({\mathcalligra {s}}\eth ({\mathscr {L}}\zeta _n,{\mathscr {L}}\zeta ))\le {\hat{\phi }}({\mathcal {C}}_{\mathcalligra {f}}(\zeta _n,\zeta ))-{\hat{\psi }}({\mathcal {D}}_{\mathcalligra {f}}(\zeta _n,\zeta )), $$

where

$$\begin{aligned} {\mathcal {C}}_{\mathcalligra {f}}(\zeta _n,\mu )&=\max \left\{\frac{\eth ({\mathcalligra {f}}\mu ,{\mathscr {L}}\mu ) \left[ 1+\eth ({\mathcalligra {f}}\zeta _n,{\mathscr {L}}\zeta _n)\right] }{1+\eth ({\mathcalligra {f}}\zeta _n,{\mathcalligra {f}}\mu )}, \frac{\eth ({\mathcalligra {f}}\zeta _n,{\mathscr {L}}\zeta _n)~\eth ({\mathcalligra {f}}\mu ,{\mathscr {L}}\mu )}{1+\eth ({\mathcalligra {f}}\zeta _n,{\mathcalligra {f}}\mu )}, \eth ({\mathcalligra {f}}\zeta _n,{\mathscr {L}}\zeta _n),\eth ({\mathcalligra {f}}\mu ,{\mathscr {L}}\mu ), \eth ({\mathcalligra {f}}\zeta _n,{\mathcalligra {f}}\mu )\right\} \\ &\rightarrow \max \{\eth ({\mathcalligra {f}}\mu ,{\mathscr {L}}\mu ),0,0,\eth ({\mathcalligra {f}}\mu ,{\mathscr {L}}\mu ),0\} \\ & = \eth ({\mathcalligra {f}}\mu ,{\mathscr {L}}\mu ) ~\text {as}~n \rightarrow +\infty , \end{aligned} $$

and

$$\begin{aligned}{\mathcal {D}}_{\mathcalligra {f}}(\zeta _n,\mu )&=\max \{\frac{\eth ({\mathcalligra {f}}\mu ,{\mathscr {L}}\mu ) \left[ 1+\eth ({\mathcalligra {f}}\zeta _n,{\mathscr {L}}\zeta _n)\right] }{1+\eth ({\mathcalligra {f}}\zeta _n,{\mathcalligra {f}}\mu )},\eth ({\mathcalligra {f}}\zeta _n,{\mathcalligra {f}}\mu )\} \\ &\rightarrow \max \{\eth ({\mathcalligra {f}}\mu ,{\mathscr {L}}\mu ),0\} \\ &= \eth ({\mathcalligra {f}}\mu ,{\mathscr {L}}\mu ) \;\text {as}\;n \rightarrow +\infty . \end{aligned} $$

Thus Eq. (23) becomes

24 $${\hat{\phi }}({\mathcalligra {s}}\lim \limits _{n \rightarrow +\infty } \eth ({\mathscr {L}}\zeta _n,{\mathscr {L}}\zeta ))\le {\hat{\phi }}(\eth ({\mathcalligra {f}}\mu ,{\mathscr {L}}\mu ))-{\hat{\psi }}(\eth ({\mathcalligra {f}}\mu ,{\mathscr {L}}\mu ))< {\hat{\phi }}(\eth ({\mathcalligra {f}}\mu ,{\mathscr {L}}\mu )).$$

As a result, we have

25 $$\lim \limits _{n \rightarrow +\infty }\eth ({\mathscr {L}}\zeta _n,{\mathscr {L}}\zeta ) < \frac{1}{{\mathcalligra {s}}}\eth ({\mathcalligra {f}}\mu ,{\mathscr {L}}\mu ).$$

Furthermore, the triangular inequality of $\eth$, we have

26 $$\frac{1}{{\mathcalligra {s}}}\eth ({\mathcalligra {f}}\mu ,{\mathscr {L}}\mu )\le \eth ({\mathcalligra {f}}\mu ,{\mathscr {L}}\zeta _n)+\eth ({\mathscr {L}}\zeta _n,{\mathscr {L}}\mu ), $$

thus Eqs. (25) and (26) lead to contradiction, if ${\mathcalligra {f}}\mu \ne {\mathscr {L}}\mu$. Hence, ${\mathcalligra {f}}\mu ={\mathscr {L}}\mu$. Let ${\mathcalligra {f}}\mu ={\mathscr {L}}\mu =\rho$, then ${\mathscr {L}}\rho = {\mathscr {L}}({\mathcalligra {f}}\mu )={\mathcalligra {f}}({\mathscr {L}}\mu )={\mathcalligra {f}}\rho$. Since ${\mathcalligra {f}}\mu ={\mathcalligra {f}}({\mathcalligra {f}}\mu )={\mathcalligra {f}}\rho$, then by Eq. (23) with ${\mathcalligra {f}}\mu ={\mathscr {L}}\mu$ and ${\mathcalligra {f}}\rho ={\mathscr {L}}\rho$, we get

27 $$ {\hat{\phi }}({\mathcalligra {s}}\eth ({\mathscr {L}}\mu ,{\mathscr {L}}\rho ))\le {\hat{\phi }}({\mathcal {C}}_{\mathcalligra {f}}(\mu ,\rho ))-{\hat{\psi }}({\mathcal {D}}_{\mathcalligra {f}}(\mu ,\rho ))<{\hat{\phi }}(\eth ({\mathscr {L}}\mu ,{\mathscr {L}}\rho )),  $$

or equivalently,

$${\mathcalligra {s}}\eth ({\mathscr {L}}\mu ,{\mathscr {L}}\rho ) \le \eth ({\mathscr {L}}\mu ,{\mathscr {L}}\rho ),$$

which is a contradiction, if ${\mathscr {L}}\mu \ne {\mathscr {L}}\rho$. Thus, ${\mathscr {L}}\mu = {\mathscr {L}}\rho = \rho$ and implies that ${\mathscr {L}}\mu = {\mathcalligra {f}}\rho =\rho$. Hence the result. $\square$

### **Definition 2.8**

Consider the partially ordered *b*-metric space $({\mathfrak {P}},\eth ,\preceq )$. A mapping ${\mathscr {L}}:{\mathfrak {P}} \times {\mathfrak {P}} \rightarrow {\mathfrak {P}}$ is a generalized $({\hat{\phi }},{\hat{\psi }})$-contractive mapping with respect to a self mapping ${\mathcalligra {f}}$ on ${\mathfrak {P}}$, if

28 $$ \phi ({\mathcalligra {s}}^k\eth ({\mathscr {L}}(\zeta ,\varpi ),{\mathscr {L}}(\varrho ,\sigma )))\le {\hat{\phi }}({\mathcal {C}}_{\mathcalligra {f}}(\zeta ,\varpi ,\varrho ,\sigma ))-{\hat{\psi }}({\mathcal {D}}_{\mathcalligra {f}}(\zeta ,\varpi ,\varrho ,\sigma )), $$

for all $\zeta ,\varpi ,\varrho ,\sigma \in {\mathfrak {P}}$ with ${\mathcalligra {f}}\zeta \preceq {\mathcalligra {f}} \varrho$ and ${\mathcalligra {f}}\varpi \succeq {\mathcalligra {f}} \sigma$, $k>2$, ${\mathcalligra {s}}>1$, ${\hat{\phi }} \in {\hat{\Phi }}$, ${\hat{\psi }} \in {\hat{\Psi }}$ and where

$$\begin{aligned} {\mathcal {C}}_{\mathcalligra {f}}(\zeta ,\varpi ,\varrho ,\sigma )&=\max \left \{\frac{\eth ({\mathcalligra {f}}\varrho ,{\mathscr {L}}(\varrho ,\sigma )) \left[ 1+\eth ({\mathcalligra {f}}\zeta ,{\mathscr {L}}(\zeta ,\varpi ))\right] }{1+\eth ({\mathcalligra {f}}\zeta ,{\mathcalligra {f}}\varrho )}, \frac{\eth ({\mathcalligra {f}}\zeta ,{\mathscr {L}}(\zeta ,\varpi ))~\eth ({\mathcalligra {f}}\varrho ,{\mathscr {L}}(\varrho ,\sigma ))}{1+\eth ({\mathcalligra {f}}\zeta ,{\mathcalligra {f}}\varrho )}, \eth ({\mathcalligra {f}}\zeta ,{\mathscr {L}}(\zeta ,\varpi )),\eth ({\mathcalligra {f}}\varrho ,{\mathscr {L}}(\varrho ,\sigma )), \eth ({\mathcalligra {f}}\zeta ,{\mathcalligra {f}}\varrho )\right\}, \end{aligned} $$

and

$$ {\mathcal {D}}_{\mathcalligra {f}}(\zeta ,\varpi ,\varrho ,\sigma )=\max \{\frac{\eth ({\mathcalligra {f}}\varrho ,{\mathscr {L}}(\varrho ,\sigma )) \left[ 1+\eth ({\mathcalligra {f}}\zeta ,{\mathscr {L}}(\zeta ,\varpi ))\right] }{1+\eth ({\mathcalligra {f}}\zeta ,{\mathcalligra {f}}\varrho )}, \eth ({\mathcalligra {f}}\zeta ,{\mathcalligra {f}}\varrho )\}. $$

### **Theorem 2.9**

*Let* $({\mathfrak {P}},\eth ,\preceq )$ *be a complete partially ordered b-metric space. Assume that a mapping* ${\mathscr {L}}:{\mathfrak {P}} \times {\mathfrak {P}} \rightarrow {\mathfrak {P}}$ *satisfies the condition (*28*) and,* ${\mathscr {L}},$ ${\mathcalligra {f}}$ *are continuous,* ${\mathscr {L}}$ *has mixed* ${\mathcalligra {f}}$*-monotone property and commutes with* ${\mathcalligra {f}}.$ *Suppose, if for some *$(\zeta _0,\varpi _0) \in {\mathfrak {P}} \times {\mathfrak {P}}$ *such that* ${\mathcalligra {f}}\zeta _0 \preceq {\mathscr {L}}(\zeta _0,\varpi _0),$ ${\mathcalligra {f}}\varpi _0 \succeq {\mathscr {L}}(\varpi _0,\zeta _0)$ *and* ${\mathscr {L}}({\mathfrak {P}} \times {\mathfrak {P}}) \subseteq {\mathcalligra {f}}({\mathfrak {P}}),$ *then* ${\mathscr {L}}$ *and* ${\mathcalligra {f}}$ *have a coupled coincidence point in* ${\mathfrak {P}}.$

### *Proof*

From Theorem 2.2 of [5], there will be two sequences $\{\zeta _n\}, \{\varpi _n\} \subset {\mathfrak {P}}$ such that

$$ {\mathcalligra {f}}\zeta _{n+1}={\mathscr {L}}(\zeta _n,\varpi _n), \quad{\mathcalligra {f}}\varpi _{n+1}={\mathscr {L}}(\varpi _n,\zeta _n),~\text {for all}~n\ge 0. $$

In particular, the sequences $\{{\mathcalligra {f}}\zeta _n\}$ and $\{{\mathcalligra {f}}\varpi _n\}$ are non-decreasing and non-increasing in ${\mathfrak {P}}$. Put $\zeta =\zeta _n, \varpi =\varpi _n, \varrho =\zeta _{n+1}, \sigma =\varpi _{n+1}$ in (28), we get

29 $$\begin{aligned} {\hat{\phi }}({\mathcalligra {s}}^k\eth ({\mathcalligra {f}}\zeta _{n+1},{\mathcalligra {f}}\zeta _{n+2}))&={\hat{\phi }}({\mathcalligra {s}}^k\eth ({\mathscr {L}}(\zeta _n,\varpi _n),{\mathscr {L}}(\zeta _{n+1},\varpi _{n+1})))\\ &\le {\hat{\phi }}({\mathcal {C}}_{\mathcalligra {f}}(\zeta _n,\varpi _n,\zeta _{n+1},\varpi _{n+1}))-{\hat{\psi }}({\mathcal {D}}_{\mathcalligra {f}}(\zeta _n,\varpi _n,\zeta _{n+1},\varpi _{n+1})), \end{aligned}$$

where

30 $$ {\mathcal {C}}_{\mathcalligra {f}}(\zeta _n,\varpi _n,\zeta _{n+1},\varpi _{n+1})\le \max \{\eth ({\mathcalligra {f}}\zeta _n,{\mathcalligra {f}}\zeta _{n+1}),\eth ({\mathcalligra {f}}\zeta _{n+1},{\mathcalligra {f}}\zeta _{n+2})\}$$

and

31 $$ {\mathcal {D}}_{\mathcalligra {f}}(\zeta _n,\varpi _n,\zeta _{n+1},\varpi _{n+1})= \max \{\eth ({\mathcalligra {f}}\zeta _n,{\mathcalligra {f}}\zeta _{n+1}),\eth ({\mathcalligra {f}}\zeta _{n+1},{\mathcalligra {f}}\zeta _{n+2})\}. $$

Therefore from (29), we have

32 $$ \begin{aligned} {\hat{\phi }}({\mathcalligra {s}}^k\eth ({\mathcalligra {f}}\zeta _{n+1},{\mathcalligra {f}}\zeta _{n+2}))& \le {\hat{\phi }}(\max \{\eth ({\mathcalligra {f}}\zeta _n,{\mathcalligra {f}}\zeta _{n+1}),\eth ({\mathcalligra {f}}\zeta _{n+1},{\mathcalligra {f}}\zeta _{n+2})\})\\ &\quad -{\hat{\psi }}(\max \{\eth ({\mathcalligra {f}}\zeta _n,{\mathcalligra {f}}\zeta _{n+1}),\eth ({\mathcalligra {f}}\zeta _{n+1},{\mathcalligra {f}}\zeta _{n+2})\}). \end{aligned}$$

Similarly by taking $\zeta =\varpi _{n+1}, \varpi =\zeta _{n+1}, \varrho =\zeta _n, \sigma =\zeta _n$ in (28), we get

33 $$ \begin{aligned} {\hat{\phi }}({\mathcalligra {s}}^k\eth ({\mathcalligra {f}}\varpi _{n+1},{\mathcalligra {f}}\varpi _{n+2}))&\le {\hat{\phi }}(\max \{\eth ({\mathcalligra {f}}\varpi _n,{\mathcalligra {f}}\varpi _{n+1}),\eth ({\mathcalligra {f}}\varpi _{n+1},{\mathcalligra {f}}\varpi _{n+2})\})\\ & \quad -{\hat{\psi }}(\max \{\eth ({\mathcalligra {f}}\varpi _n,{\mathcalligra {f}}\varpi _{n+1}),\eth ({\mathcalligra {f}}\varpi _{n+1},{\mathcalligra {f}}\varpi _{n+2})\}). \end{aligned}$$

We know that $\max \{{\hat{\phi }}(\varepsilon _1),{\hat{\phi }}(\varepsilon _2)\}={\hat{\phi }} \{\max \{\varepsilon _1,\varepsilon _2\}\}$ for $\varepsilon _1,\varepsilon _2 \in [0,+\infty )$. Then by adding Eqs. (32) and (33) together to get,

34 $$ \begin{aligned} {\hat{\phi }}({\mathcalligra {s}}^k \delta _n)&\le \phi (\max \{\eth ({\mathcalligra {f}}\zeta _n,{\mathcalligra {f}}\zeta _{n+1}),\eth ({\mathcalligra {f}}\zeta _{n+1},{\mathcalligra {f}}\zeta _{n+2}),\eth ({\mathcalligra {f}}\varpi _n,{\mathcalligra {f}}\varpi _{n+1}),\eth ({\mathcalligra {f}}\varpi _{n+1},{\mathcalligra {f}}\varpi _{n+2})\})\\ & \quad -{\hat{\psi }}(\max \{\eth ({\mathcalligra {f}}\zeta _n,{\mathcalligra {f}}\zeta _{n+1}),\eth ({\mathcalligra {f}}\zeta _{n+1},{\mathcalligra {f}}\zeta _{n+2}),\eth ({\mathcalligra {f}}\varpi _n,{\mathcalligra {f}}\varpi _{n+1}),\eth ({\mathcalligra {f}}\varpi _{n+1},{\mathcalligra {f}}\varpi _{n+2})\}) \end{aligned} $$

where

35 $$\delta _n=\max \{\eth ({\mathcalligra {f}}\zeta _{n+1},{\mathcalligra {f}}\zeta _{n+2}), \eth ({\mathcalligra {f}}\varpi _{n+1},{\mathcalligra {f}}\varpi _{n+2})\}.$$

Let us denote,

36 $$ \nabla _n=\max \{\eth ({\mathcalligra {f}}\zeta _n,{\mathcalligra {f}}\zeta _{n+1}),\eth ({\mathcalligra {f}}\zeta _{n+1},{\mathcalligra {f}}\zeta _{n+2}),\eth ({\mathcalligra {f}}\varpi _n,{\mathcalligra {f}}\varpi _{n+1}),\eth ({\mathcalligra {f}}\varpi _{n+1},{\mathcalligra {f}}\varpi _{n+2})\}. $$

Hence from Eqs. (32)–(35), we obtain that

37 $${\mathcalligra {s}}^k\delta _n\le \nabla _n.$$

Now to claim that

38 $$ \delta _n\le \lambda \delta _{n-1}, $$

for $$n \ge 1$$ and $$\lambda =\frac{1}{{\mathcalligra {s}}^k} \in [0,1)$$.

Suppose that if $\nabla _n=\delta _n$ then from (37), we get ${\mathcalligra {s}}^k\delta _n\le \delta _n$ this leads to $\delta _n=0$ since ${\mathcalligra {s}}>1$ and thus (38) holds. Suppose $\nabla _n=\max \{\eth ({\mathcalligra {f}}\zeta _n,{\mathcalligra {f}}\zeta _{n+1}), \eth ({\mathcalligra {f}}\varpi _n,{\mathcalligra {f}}\varpi _{n+1})\}$, that is, $\nabla _n=\delta _{n-1}$ thence (37) follows (38).

Now, we can deduce from (37) that $\delta _n\le \lambda ^n \delta _0$ and therefore,

39 $$ \eth ({\mathcalligra {f}}\zeta _{n+1},{\mathcalligra {f}}\zeta _{n+2})\le \lambda ^n \delta _0 ~~\text {and}~~\eth ({\mathcalligra {f}}\varpi _{n+1},{\mathcalligra {f}}\varpi _{n+2})\le \lambda ^n \delta _0,$$

which shows that $\{{\mathcalligra {f}}\zeta _n\}$ and $\{{\mathcalligra {f}}\varpi _n\}$ in ${\mathfrak {P}}$ are Cauchy sequences from Lemma 3.1 of [15]. Therefore, we can conclude from [3] of Theorem 2.2 that ${\mathscr {L}}$ and ${\mathcalligra {f}}$ in ${\mathfrak {P}}$ have a coincidence point. $\square$

### **Corollary 2.10**

*Suppose* $({\mathfrak {P}},\eth ,\preceq )$ *be a complete partially ordered* *b-metric space. Let a continuous mapping* ${\mathscr {L}}:{\mathfrak {P}} \times {\mathfrak {P}} \rightarrow {\mathfrak {P}}$ *has a mixed monotone property and satisfies the contraction conditions below for any* $\zeta ,\varpi ,\varrho ,\sigma \in {\mathfrak {P}}$ *such that* $\zeta \preceq \varrho$ *and* $\varpi \succeq \sigma,$ $k>2,$ ${\mathcalligra {s}}>1,$ ${\hat{\phi }} \in {\hat{\Phi }}$ *and* ${\hat{\psi }} \in {\hat{\Psi }}:$

i. $$ {\hat{\phi }}({\mathcalligra {s}}^k\eth ({\mathscr {L}}(\zeta ,\varpi ),{\mathscr {L}}(\varrho ,\sigma )))\le {\hat{\phi }}({\mathcal {C}}_{\mathcalligra {f}}(\zeta ,\varpi ,\varrho ,\sigma ))-{\hat{\psi }}({\mathcal {D}}_{\mathcalligra {f}}(\zeta ,\varpi ,\varrho ,\sigma )), $$
ii. $$ \eth ({\mathscr {L}}(\zeta ,\varpi ),{\mathscr {L}}(\varrho ,\sigma ))\le \frac{1}{{\mathcalligra {s}}^k}{\mathcal {C}}_{\mathcalligra {f}}(\zeta ,\varpi ,\varrho ,\sigma )-\frac{1}{{\mathcalligra {s}}^k}{\hat{\psi }}({\mathcal {D}}_{\mathcalligra {f}}(\zeta ,\varpi ,\varrho ,\sigma )).$$

*where*

$${\mathcal {C}}_{\mathcalligra {f}}(\zeta ,\varpi ,\varrho ,\sigma ) =\max \left\{\frac{\eth (\varrho ,{\mathscr {L}}(\varrho ,\sigma )) \left[ 1+\eth (\zeta ,{\mathscr {L}}(\zeta ,\varpi ))\right] }{1+\eth (\zeta ,\varrho )}, \frac{\eth (\zeta ,{\mathscr {L}}(\zeta ,\varpi ))\;\eth (\varrho ,{\mathscr {L}}(\varrho ,\sigma ))}{1+\eth (\zeta ,\varrho )},\eth (\zeta ,{\mathscr {L}}(\zeta ,\varpi )),\eth (\varrho ,{\mathscr {L}}(\varrho ,\sigma )), \eth (\zeta ,\varrho )\right\}, $$

*and*

$${\mathcal {D}}_{\mathcalligra {f}}(\zeta ,\varpi ,\varrho ,\sigma )=\max \{\frac{\eth (\varrho ,{\mathscr {L}}(\varrho ,\sigma )) \left[ 1+\eth (\zeta ,{\mathscr {L}}(\zeta ,\varpi ))\right] }{1+\eth (\zeta ,\varrho )}, \eth (\zeta ,\varrho )\}. $$

*If there exists* $(\zeta _0,\varpi _0) \in {\mathfrak {P}} \times {\mathfrak {P}}$ *such that* $\zeta _0 \preceq {\mathscr {L}}(\zeta _0,\varpi _0)$ *and* $\varpi _0 \succeq {\mathscr {L}}(\varpi _0,\zeta _0),$ *then* ${\mathscr {L}}$ *has a coupled fixed point in* ${\mathfrak {P}}.$

### **Theorem 2.11**

*A unique coupled common fixed point for* ${\mathscr {L}}$ *and* ${\mathcalligra {f}}$ *exists in Theorem* 2.9*, if for every* $(\zeta ,\varpi ),({\mathcalligra {k}},{\mathcalligra {l}}) \in {\mathfrak {P}} \times {\mathfrak {P}}$ *there is some* $(\mathscr {\alpha}^*,\mathscr {\beta}^*)\in {\mathfrak {P}} \times {\mathfrak {P}}$ *such that* $({\mathscr {L}}(\mathscr {\alpha}^*,\mathscr {\beta}^*), {\mathscr {L}}(\mathscr {\beta}^*,\mathscr {\alpha}^*))$ *is comparable to* $({\mathscr {L}}(\zeta ,\varpi ), {\mathscr {L}}(\varpi ,\zeta ))$ *and to* $({\mathscr {L}}({\mathcalligra {k}},{\mathcalligra {l}}),{\mathscr {L}}({\mathcalligra {l}},{\mathcalligra {k}})).$

### *Proof*

From Theorem 2.9, the mappings ${\mathscr {L}}$ and ${\mathcalligra {f}}$ have a coupled coincidence point in ${\mathfrak {P}}$. Let $(\zeta , \varpi ),({\mathcalligra {k}},{\mathcalligra {l}}) \in {\mathfrak {P}} \times {\mathfrak {P}}$ are two coupled coincidence points of ${\mathscr {L}}$ and ${\mathcalligra {f}}$. Now to claim that ${\mathcalligra {f}}\zeta ={\mathcalligra {f}}{\mathcalligra {k}}$ and ${\mathcalligra {f}}\varpi ={\mathcalligra {f}}{\mathcalligra {l}}$. By hypotheses $({\mathscr {L}}(\mathscr {\alpha}^*,\mathscr {\beta}^*), {\mathscr {L}}(\mathscr {\beta}^*,\mathscr {\alpha}^*))$ is comparable to $({\mathscr {L}}(\zeta ,\varpi ), {\mathscr {L}}(\varpi ,\zeta ))$ for some $(\mathscr {\alpha}^*,\mathscr {\beta}^*)\in {\mathfrak {P}} \times {\mathfrak {P}}$.

Now, assume the following

$$\begin{aligned}& ({\mathscr {L}}(\zeta ,\varpi ), {\mathscr {L}}(\varpi ,\zeta )) \le ({\mathscr {L}}(\mathscr {\alpha}^*,\mathscr {\beta}^*), {\mathscr {L}}(\mathscr {\beta}^*,\mathscr {\alpha}^*))~ \text {and} \\ & ({\mathscr {L}}({\mathcalligra {k}},{\mathcalligra {l}}),{\mathscr {L}}({\mathcalligra {l}},{\mathcalligra {k}}))\le ({\mathscr {L}}(\mathscr {\alpha}^*,\mathscr {\beta}^*), {\mathscr {L}}(\mathscr {\beta}^*,\mathscr {\alpha}^*)). \end{aligned}$$

Suppose $\mathscr {\alpha}^*_0=\mathscr {\alpha}^*$ and $\mathscr {\beta}^*_0=\mathscr {\beta}^*$ then there is a point $(\mathscr {\alpha}^*_1,\mathscr {\beta}^*_1) \in {\mathfrak {P}} \times {\mathfrak {P}}$ such that

$${\mathcalligra {f}}\mathscr {\alpha}^*_1={\mathscr {L}}(\mathscr {\alpha}^*_0,\mathscr {\beta}^*_0),\; {\mathcalligra {f}}\mathscr {\beta}^*_1={\mathscr {L}}(\mathscr {\beta}^*_0,\mathscr {\alpha}^*_0)~~(n \ge 1).$$

We have the sequences $\{{\mathcalligra {f}} \mathscr {\alpha}^*_{n}\}$ and $\{{\mathcalligra {f}} \mathscr {\beta}^*_{n}\}$ in ${\mathfrak {P}}$ as by the repeated application of the above argument with

$$ {\mathcalligra {f}}\mathscr {\alpha}^*_{n+1}={\mathscr {L}}(\mathscr {\alpha}^*_n,\mathscr {\beta}^*_n),\; {\mathcalligra {f}}\mathscr {\beta}^*_{n+1}={\mathscr {L}}(\mathscr {\beta}^*_n,\mathscr {\alpha}^*_n),~n \ge 0. $$

Similarly, define the sequences $\{{\mathcalligra {f}} \zeta _{n}\}$, $\{{\mathcalligra {f}} \varpi _{n}\}$ and $\{{\mathcalligra {f}} {\mathcalligra {k}}_{n}\}$, $\{{\mathcalligra {f}} {\mathcalligra {l}}_{n}\}$ in ${\mathfrak {P}}$ by setting $\zeta _0=\zeta$, $\varpi _0=\varpi$ and ${\mathcalligra {k}}_0={\mathcalligra {k}}$, ${\mathcalligra {l}}_0={\mathcalligra {l}}$. Furthermore, we have

40 $${\mathcalligra {f}}\zeta _{n} \rightarrow {\mathscr {L}}(\zeta ,\varpi ),\;{\mathcalligra {f}}\varpi _{n} \rightarrow {\mathscr {L}}(\varpi ,\zeta ),\; {\mathcalligra {f}}{\mathcalligra {k}}_{n} \rightarrow {\mathscr {L}}({\mathcalligra {k}},{\mathcalligra {l}}),\;{\mathcalligra {f}}{\mathcalligra {l}}_n \rightarrow {\mathscr {L}}({\mathcalligra {l}},{\mathcalligra {k}})\;(n \ge 1).$$

Therefore by induction, we have

41 $$ ({\mathcalligra {f}}\zeta _{n},{\mathcalligra {f}}\varpi _{n}) \le ({\mathcalligra {f}}\mathscr {\alpha}^*_n,{\mathcalligra {f}}\mathscr {\beta}^*_n),\;n\ge 0. $$

Now from Eq. (28), we get

42 $$ \begin{aligned} {\hat{\phi }}(\eth ({\mathcalligra {f}}\zeta ,{\mathcalligra {f}}\mathscr {\alpha}^*_{n+1}))& \le {\hat{\phi }}({\mathcalligra {s}}^k\eth ({\mathcalligra {f}}\zeta ,{\mathcalligra {f}}\mathscr {\alpha}^*_{n+1}))= {\hat{\phi }}({\mathcalligra {s}}^k\eth ({\mathscr {L}}(\zeta ,\varpi ),{\mathscr {L}}(\mathscr {\alpha}^*_n,\mathscr {\beta}^*_n))) \\ &\le {\hat{\phi }}({\mathcal {C}}_{\mathcalligra {f}}(\zeta ,\varpi ,\mathscr {\alpha}^*_n,\mathscr {\beta}^*_n))-{\hat{\psi }}({\mathcal {D}}_{\mathcalligra {f}}(\zeta ,\varpi ,\mathscr {\alpha}^*_n,\mathscr {\beta}^*_n)), \end{aligned}$$

where

$$\begin{aligned} {\mathcal {C}}_{\mathcalligra {f}}(\zeta ,\varpi ,\mathscr {\alpha}^*_n,\mathscr {\beta}^*_n)& =\max \left\{\frac{\eth ({\mathcalligra {f}}\mathscr {\alpha}^*_n,{\mathscr {L}}(\mathscr {\alpha}^*_n,\mathscr {\beta}^*_n)) \left[ 1+\eth ({\mathcalligra {f}}\zeta ,{\mathscr {L}}(\zeta ,\varpi ))\right] }{1+\eth ({\mathcalligra {f}}\zeta ,{\mathcalligra {f}}\mathscr {\alpha}^*_n)}, \frac{\eth ({\mathcalligra {f}}\zeta ,{\mathscr {L}}(\zeta ,\varpi ))\;\eth ({\mathcalligra {f}}\mathscr {\alpha}^*_n,{\mathscr {L}}(\mathscr {\alpha}^*_n,\mathscr {\beta}^*_n))}{1+\eth ({\mathcalligra {f}}\zeta ,{\mathcalligra {f}}\mathscr {\alpha}^*_n)}, \eth ({\mathcalligra {f}}\zeta ,{\mathscr {L}}(\zeta ,\varpi )),\eth ({\mathcalligra {f}}\mathscr {\alpha}^*_n,{\mathscr {L}}(\mathscr {\alpha}^*_n,\mathscr {\beta}^*_n)), \eth ({\mathcalligra {f}}\zeta ,{\mathcalligra {f}}\mathscr {\alpha}^*_n)\right\} \\ & = \max \{0,\eth ({\mathcalligra {f}}\zeta ,{\mathcalligra {f}}\mathscr {\alpha}^*_n)\} \\ & =\eth ({\mathcalligra {f}}\zeta ,{\mathcalligra {f}}\mathscr {\alpha}^*_n) \end{aligned} $$

and

$$ \begin{aligned} {\mathcal {D}}_{\mathcalligra {f}}(\zeta ,\varpi ,\mathscr {\alpha}^*_n,\mathscr {\beta}^*_n)&=\max \left\{\frac{\eth ({\mathcalligra {f}}\mathscr {\alpha}^*_n,{\mathscr {L}}(\mathscr {\alpha}^*_n,\mathscr {\beta}^*_n)) \left[ 1+\eth ({\mathcalligra {f}}\zeta ,{\mathscr {L}}(\zeta ,\varpi ))\right] }{1+\eth ({\mathcalligra {f}}\zeta ,f\mathscr {\alpha}^*_n)}, \eth ({\mathcalligra {f}}\zeta ,f\mathscr {\alpha}^*_n)\right\} \\ &=\eth ({\mathcalligra {f}}\zeta ,{\mathcalligra {f}}\mathscr {\alpha}^*_n). \end{aligned}$$

As a result of Eq. (42), we now have

43 $$ {\hat{\phi }}(\eth ({\mathcalligra {f}}\zeta ,{\mathcalligra {f}}\mathscr {\alpha}^*_{n+1}))\le {\hat{\phi }}(\eth ({\mathcalligra {f}}\zeta ,{\mathcalligra {f}}\mathscr {\alpha}^*_n))-{\hat{\psi }}(\eth ({\mathcalligra {f}}\zeta ,{\mathcalligra {f}}\mathscr {\alpha}^*_n)). $$

As a consequence of a similar argument, we deduce that

44 $$ {\hat{\phi }}(\eth ({\mathcalligra {f}}\varpi ,{\mathcalligra {f}}\mathscr {\beta}^*_{n+1}))\le {\hat{\phi }}(\eth ({\mathcalligra {f}}\varpi ,{\mathcalligra {f}}\mathscr {\beta}^*_n))-{\hat{\psi }}(\eth ({\mathcalligra {f}}\varpi ,{\mathcalligra {f}}\mathscr {\beta}^*_n)). $$

Therefore from (43) and (44), we have

45 $$ \begin{aligned} {\hat{\phi }}(\max \{\eth ({\mathcalligra {f}}\zeta ,{\mathcalligra {f}}\mathscr {\alpha}^*_{n+1}),\eth ({\mathcalligra {f}}\varpi ,{\mathcalligra {f}}\mathscr {\beta}^*_{n+1})\})&\le {\hat{\phi }}(\max \{\eth ({\mathcalligra {f}}\zeta ,{\mathcalligra {f}}\mathscr {\alpha}^*_n),\eth ({\mathcalligra {f}}\varpi ,{\mathcalligra {f}}\mathscr {\beta}^*_n)\})\\ & \quad -{\hat{\psi }}(\max \{\eth ({\mathcalligra {f}}\zeta ,{\mathcalligra {f}}\mathscr {\alpha}^*_n),\eth ({\mathcalligra {f}}\varpi ,{\mathcalligra {f}}\mathscr {\beta}^*_n)\}) \\ & <{\hat{\phi }}(\max \{\eth ({\mathcalligra {f}}\zeta ,{\mathcalligra {f}}\mathscr {\alpha}^*_n),\eth ({\mathcalligra {f}}\varpi ,{\mathcalligra {f}}\mathscr {\beta}^*_n)\}).  \end{aligned}$$

The property of ${\hat{\phi }}$ implies that,

$$ \max \{\eth ({\mathcalligra {f}}\zeta ,{\mathcalligra {f}}\mathscr {\alpha}^*_{n+1}),\eth ({\mathcalligra {f}}\varpi ,{\mathcalligra {f}}\mathscr {\beta}^*_{n+1})\} <\max \{\eth ({\mathcalligra {f}}\zeta ,{\mathcalligra {f}}\mathscr {\alpha}^*_n),\eth ({\mathcalligra {f}}\varpi ,{\mathcalligra {f}}\mathscr {\beta}^*_n)\}. $$

Hence, $\max \{\eth ({\mathcalligra {f}}\zeta ,{\mathcalligra {f}}\mathscr {\alpha}^*_n),\eth ({\mathcalligra {f}}\varpi ,{\mathcalligra {f}}\mathscr {\beta}^*_n)\}$ is bounded below decreasing sequence of positive reals and by a result, we get

$$\lim \limits _{n \rightarrow +\infty }\max \{\eth ({\mathcalligra {f}}\zeta ,{\mathcalligra {f}}\mathscr {\alpha}^*_n),\eth ({\mathcalligra {f}}\varpi ,{\mathcalligra {f}}\mathscr {\beta}^*_n)\} =\Gamma ,\;\Gamma \ge 0. $$

Therefore as $n \rightarrow +\infty$ in Eq. (45), we get

46 $${\hat{\phi }}(\Gamma )\le {\hat{\phi }}(\Gamma )-{\hat{\psi }}(\Gamma ), $$

which we have derived ${\hat{\psi }}(\Gamma )=0$. Hence, $\Gamma =0$. Therefore,

$$ \lim \limits _{n \rightarrow +\infty }\max \{\eth ({\mathcalligra {f}}\zeta ,{\mathcalligra {f}}\mathscr {\alpha}^*_n),\eth ({\mathcalligra {f}}\varpi ,{\mathcalligra {f}}\mathscr {\beta}^*_n)\} =0. $$

Thus,

47 $$ \lim \limits _{n \rightarrow +\infty }\eth ({\mathcalligra {f}}\zeta ,{\mathcalligra {f}}\mathscr {\alpha}^*_n) =0 ~ \text {and} ~\lim \limits _{n \rightarrow +\infty }\eth ({\mathcalligra {f}}\varpi ,{\mathcalligra {f}}\mathscr {\beta}^*_n) =0. $$

Also from the above same argument, we procured that

48 $$ \lim \limits _{n \rightarrow +\infty }\eth ({\mathcalligra {f}}{\mathcalligra {k}},{\mathcalligra {f}}\mathscr {\alpha}^*_n) =0 ~ \text {and} ~\lim \limits _{n \rightarrow +\infty }\eth ({\mathcalligra {f}}{\mathcalligra {l}},{\mathcalligra {f}}\mathscr {\beta}^*_n) =0. $$

Therefore from (47) and (48), we get ${\mathcalligra {f}}\zeta ={\mathcalligra {f}}{\mathcalligra {k}}$ and ${\mathcalligra {f}}\varpi ={\mathcalligra {f}}{\mathcalligra {l}}$. Since ${\mathcalligra {f}}\zeta ={\mathscr {L}}(\zeta ,\varpi )$ and ${\mathcalligra {f}}\varpi ={\mathscr {L}}(\varpi ,\zeta )$ and the commutativity property of ${\mathscr {L}}$ and ${\mathcalligra {f}}$ implies that

49 $$ {\mathcalligra {f}}({\mathcalligra {f}}\zeta )= {\mathcalligra {f}}({\mathscr {L}}(\zeta ,\varpi ))={\mathscr {L}}({\mathcalligra {f}}\zeta ,{\mathcalligra {f}}\varpi )~ \text {and}~{\mathcalligra {f}}({\mathcalligra {f}}\varpi )= {\mathcalligra {f}}({\mathscr {L}}(\varpi ,\zeta ))={\mathscr {L}}({\mathcalligra {f}}\varpi ,{\mathcalligra {f}}\zeta ). $$

If ${\mathcalligra {f}}\zeta =\mathscr {{\alpha}^*}_1$ and ${\mathcalligra {f}}\varpi =\mathscr {{\beta}^*}_1$ then from (49), we get

50 $$ {\mathcalligra {f}}(\mathscr {{\alpha}^*}_1)= {\mathscr {L}}(\mathscr {{\alpha}^*}_1,\mathscr {{\beta}^*}_1)~ \text {and}~{\mathcalligra {f}}(\mathscr {{\beta}^*}_1)= {\mathscr {L}}(\mathscr {{\beta}^*}_1,\mathscr {{\alpha}^*}_1), $$

this shows that $(\mathscr {{\alpha}^*}_1,\mathscr {{\beta}^*}_1)$ is a coupled coincidence point of ${\mathscr {L}}$ and ${\mathcalligra {f}}$. Hence, ${\mathcalligra {f}}(\mathscr {{\alpha}^*}_1)={\mathcalligra {f}}{\mathcalligra {k}}$ and ${\mathcalligra {f}}(\mathscr {{\beta}^*}_1)={\mathcalligra {f}}{\mathcalligra {l}}$ which in turn gives that ${\mathcalligra {f}}(\mathscr {{\alpha}^*}_1)=\mathscr {{\alpha}^*}_1$ and ${\mathcalligra {f}}(\mathscr {{\beta}^*}_1)=\mathscr {{\beta}^*}_1$. Therefore, we conclude from (50) that $(\mathscr {{\alpha}^*}_1,\mathscr {{\beta}^*}_1)$ is a coupled common fixed point of ${\mathscr {L}}$ and ${\mathcalligra {f}}$.

Assume $(\mathscr {{\alpha}^*}_2,\mathscr {{\beta}^*}_2)$ is another coupled common fixed point to ${\mathscr {L}}$ and ${\mathcalligra {f}}$. Thus $\mathscr {{\alpha}^*}_2={\mathcalligra {f}}\mathscr {{\alpha}^*}_2= {\mathscr {L}}(\mathscr {{\alpha}^*}_2,\mathscr {{\beta}^*}_2)$ and $\mathscr {{\beta}^*}_2={\mathcalligra {f}}\mathscr {{\beta}^*}_2= {\mathscr {L}}(\mathscr {{\beta}^*}_2,\mathscr {{\alpha}^*}_2)$. But $(\mathscr {{\alpha}^*}_2,\mathscr {{\beta}^*}_2)$ is a coupled common fixed point of ${\mathscr {L}}$ and ${\mathcalligra {f}}$ then ${\mathcalligra {f}}\mathscr {{\alpha}^*}_2={\mathcalligra {f}}\zeta =\mathscr {{\alpha}^*}_1$ and ${\mathcalligra {f}}\mathscr {{\beta}^*}_2={\mathcalligra {f}}\varpi =\mathscr {{\beta}^*}_1$. Therefore, $\mathscr {{\alpha}^*}_2={\mathcalligra {f}}\mathscr {{\alpha}^*}_2={\mathcalligra {f}}\mathscr {{\alpha}^*}_1=\mathscr {{\alpha}^*}_1$ and $\mathscr {{\beta}^*}_2={\mathcalligra {f}}\mathscr {{\beta}^*}_2={\mathcalligra {f}}\mathscr {{\beta}^*}_1=\mathscr {{\beta}^*}_1$. Hence the uniqueness. $\square$

### **Theorem 2.12**

*If* ${\mathcalligra {f}}\zeta _0 \preceq {\mathcalligra {f}}\varpi _0$ *or* ${\mathcalligra {f}}\zeta _0 \succeq {\mathcalligra {f}}\varpi _0$ *in Theorem* 2.11*, then* ${\mathscr {L}}$ *and* ${\mathcalligra {f}}$ *have a unique common fixed point in* ${\mathfrak {P}}.$

### *Proof*

Assume that $(\zeta ,\varpi ) \in {\mathfrak {P}}$ is a unique coupled common fixed point of ${\mathscr {L}}$ and ${\mathcalligra {f}}$. Next to show that $\zeta =\varpi$. Suppose that ${\mathcalligra {f}}\zeta _0 \preceq {\mathcalligra {f}}\varpi _0$ then by induction, we get ${\mathcalligra {f}}\zeta _n \preceq {\mathcalligra {f}}\varpi _n$ for all $n \ge 0$. From Lemma 2 of [16], we have

$$\begin{aligned} {\hat{\phi }}\left({\mathcalligra {s}}^{k-2}\eth (\zeta ,\varpi )\right)& ={\hat{\phi }}({\mathcalligra {s}}^k \frac{1}{{\mathcalligra {s}}^2}\eth (\zeta ,\varpi )) \le \lim \limits _{n \rightarrow +\infty }\sup {\hat{\phi }}({\mathcalligra {s}}^k \eth (\zeta _{n+1}, \varpi _{n+1})) \\ & = \lim \limits _{n \rightarrow +\infty }\sup {\hat{\phi }}({\mathcalligra {s}}^k \eth ({\mathscr {L}}(\zeta _n, \varpi _n),{\mathscr {L}}(\varpi _n,\zeta _n))) \\ & \le \lim \limits _{n \rightarrow +\infty }\sup {\hat{\phi }}({\mathcal {C}}_{\mathcalligra {f}}(\zeta _n, \varpi _n,\varpi _n,\zeta _n))-\lim \limits _{n \rightarrow +\infty }\inf {\hat{\psi }}({\mathcal {D}}_{\mathcalligra {f}}(\zeta _n, \varpi _n,\varpi _n,\zeta _n)) \\ & \le {\hat{\phi }}(\eth (\zeta ,\varpi ))-\lim \limits _{n \rightarrow +\infty }\inf {\hat{\psi }}({\mathcal {D}}_f(\zeta _n, \varpi _n,\varpi _n,\zeta _n)) \\ & <{\hat{\phi }}(\eth (\zeta ,\varpi )), \end{aligned}$$

which is a contradiction. Hence, $\zeta =\varpi$.

The result can also be seen in the case of ${\mathcalligra {f}}\zeta _0 \succeq {\mathcalligra {f}}\varpi _0$. $\square$

### **Note 1**

The same conclusions can also be seen as in Theorems  2.6, 2.7, 2.9,  2.11 and  2.12 by maintaining only ${\mathcal {C}}_{\mathcalligra {f}}(\zeta ,\varpi )$, ${\mathcal {C}}_{\mathcalligra {f}}(\zeta ,\varpi , \varrho , \sigma )$ in place of ${\mathcal {D}}_{\mathcalligra {f}}(\zeta ,\varpi )$, ${\mathcal {D}}_{\mathcalligra {f}}(\zeta ,\varpi , \varrho , \sigma )$ in the contraction conditions.

### *Remark 2.13*

Although ${\mathcalligra {s}}=1$ and as a consequence of [14], the condition

$$ {\hat{\phi }}(\eth ({\mathscr {L}}(\zeta ,\varpi ),{\mathscr {L}}(\varrho ,\varpi ))) \le {\hat{\phi }}(\max \{\eth ({\mathcalligra {f}}\zeta ,{\mathcalligra {f}}\varrho ),\eth ({\mathcalligra {f}}\varpi ,{\mathcalligra {f}}\varpi )\})-{\hat{\psi }}(\max \{\eth ({\mathcalligra {f}}\zeta ,{\mathcalligra {f}}\varrho ),\eth ({\mathcalligra {f}}\varpi ,{\mathcalligra {f}}\varpi )\}) $$

is equivalent to,

$$ \eth ({\mathscr {L}}(\zeta ,\varpi ),{\mathscr {L}}(\varrho ,\varpi ))\le \varphi (\max \{\eth ({\mathcalligra {f}}\zeta ,{\mathcalligra {f}}\varrho ),\eth ({\mathcalligra {f}}\varpi ,{\mathcalligra {f}}\varpi )\}), $$

where ${\hat{\phi }} \in {\hat{\Phi }}$, ${\hat{\psi }} \in {\hat{\Psi }}$ and $\varphi$ is a continuous self mapping on $[0,+\infty )$ with $\varphi (\varepsilon )<\varepsilon$ for all $\varepsilon >0$ and $\varphi (\varepsilon )=0$ if and only if $\varepsilon =0$. As a result, the findings are generalized and expanded the results of [9, 12, 17] as well as several other comparable results.

Now depending on the continuity of a metric $\eth$, we have the following examples.

### *Example 2.14*

Let ${\mathfrak {P}}=\{ a,b,c,d,e,f \}$ and $\eth :{\mathfrak {P}} \times {\mathfrak {P}} \rightarrow {\mathfrak {P}}$ be a metric defined by

$$\begin{aligned} \eth (\zeta , \varpi )& =\eth (\varpi ,\zeta )=0, \;\text {if}\; \zeta = \varpi = a,b,c,d,e,f\;\text {and}\; \zeta =\varpi ,\\ \eth (\zeta , \varpi )& =\eth (\varpi ,\zeta )=3, \;if\; \zeta = \varpi = a,b,c,d,e \;\text {and}\; \zeta \ne \varpi ,\\ \eth (\zeta , \varpi ) &=\eth (\varpi ,\zeta )=12, \;if\; \zeta = a,b,c,d \;\text {and}\; \varpi = f ,\\ \eth (\zeta , \varpi ) &=\eth (\varpi ,\zeta )=20, \;if\; \zeta = e \;\text {and}\; \varpi = f,\; \text {with}\;\text {usual}\;\text {order}\;\le .\ \end{aligned}$$

A self mapping ${\mathscr {L}}$ on ${\mathfrak {P}}$ defined by ${\mathscr {L}} a ={\mathscr {L}} b ={\mathscr {L}} c ={\mathscr {L}} d ={\mathscr {L}} e =1, {\mathscr {L}} f =2$ has a fixed point with ${\hat{\phi }}(\varepsilon )=\frac{\varepsilon }{2}$ and ${\hat{\psi }}(\varepsilon )=\frac{\varepsilon }{4}$ where $\varepsilon \in [0,+\infty )$.

### *Proof*

For ${\mathcalligra {s}}=2$, $({\mathfrak {P}},\eth ,\le )$ is a complete partially ordered *b*-metric space. Assume that $\zeta < \varpi$ for $\zeta , \varpi \in {\mathfrak {P}}$, then we have the following cases:

### **Case 1**

If $\zeta , \varpi \in \{ a,b,c,d,e \}$ then $\eth ({\mathscr {L}}\zeta ,{\mathscr {L}}\varpi )=\eth ( a , a )=0$. Thus,

$${\hat{\phi }}(2\eth ({\mathscr {L}}\zeta ,{\mathscr {L}}\varpi ))=0 \le {\hat{\phi }}({\mathcal {C}}(\zeta ,\varpi ))-{\hat{\psi }}({\mathcal {D}}(\zeta ,\varpi )). $$

### **Case 2**

If $\zeta \in \{ a,b,c,d,e \}$ and $\varpi = f$, then $\eth ({\mathscr {L}}\zeta ,{\mathscr {L}}\varpi )=\eth ( a , b )=3$, ${\mathcal {C}}( f , e )={\mathcal {D}}( f , e )=20$ and ${\mathcal {C}}(\zeta , f )={\mathcal {D}}(\zeta , f )=12$, for $\zeta \in \{ a,b,c,d \}$. Hence,

$${\hat{\phi }}(2\eth ({\mathscr {L}}\zeta ,{\mathscr {L}}\varpi )) \le \frac{{\mathcal {C}}(\zeta ,\varpi )}{ d } ={\hat{\phi }}({\mathcal {C}}(\zeta ,\varpi ))-{\hat{\psi }}({\mathcal {D}}(\zeta ,\varpi )).$$

As a result, all the conditions of Theorem 2.1 are satisfied, and hence ${\mathscr {L}}$ has a fixed point in ${\mathfrak {P}}$. $\square$

### *Example 2.15*

Define a metric $\eth$ with usual order $\le$ by

$$ \eth (\zeta ,\varpi )= {\left\{ \begin{array}{ll}  0, & if\; \zeta =\varpi \\ 1,& if\; \zeta \ne \varpi \in \{0,1\} \\ |\zeta -\varpi |,& if\; \zeta ,\varpi \in \left\{0, \frac{1}{2n},\frac{1}{2m}: n \ne m \ge 1\right\} \\ 6,& otherwise. \end{array}\right. }  $$

where ${\mathfrak {P}}=\{0, 1, \frac{1}{2},\frac{1}{3},\frac{1}{4},\cdots\frac{1}{n},\cdots\}$. Then a self mapping ${\mathscr {L}}$ on ${\mathfrak {P}}$ by ${\mathscr {L}}0=0, {\mathscr {L}}\frac{1}{n}=\frac{1}{12n} (n\ge 1)$ has a fixed point with ${\hat{\phi }}(\varepsilon )=\varepsilon$ and ${\hat{\psi }}(\varepsilon )=\frac{4\varepsilon }{5}$ for $\varepsilon \in [0,+\infty )$.

### *Proof*

$\eth$ is evidently discontinuous, and $({\mathfrak {P}},\eth ,\le )$ is a complete partially ordered *b*-metric space with ${\mathcalligra {s}}=\frac{12}{5}$. Now we have the following cases for $\zeta ,\varpi \in {\mathfrak {P}}$ with $\zeta <\varpi$:

### **Case 1**

Suppose $\zeta =0$ and $\varpi =\frac{1}{n} ~(n >0)$, then $\eth ({\mathscr {L}}\zeta ,{\mathscr {L}}\varpi )=\eth (0,\frac{1}{12n})=\frac{1}{12n}$ and ${\mathcal {C}}(\zeta ,\varpi )~\text {or}~ {\mathcal {D}}(\zeta ,\varpi )=\frac{1}{n}$ and ${\mathcal {C}}(\zeta ,\varpi )~ \text {or}~ {\mathcal {D}}(\zeta ,\varpi )= \{1,6\}$. Therefore,

$$ {\hat{\phi }}\left( \frac{12}{5}\eth ({\mathscr {L}}\zeta ,{\mathscr {L}}\varpi )\right) \le \frac{{\mathcal {C}}(\zeta ,\varpi )}{5} ={\hat{\phi }}({\mathcal {C}}(\zeta ,\varpi ))-{\hat{\psi }}({\mathcal {D}}(\zeta ,\varpi )).$$

### **Case 2**

Suppose that $\zeta =\frac{1}{m}$ and $\varpi =\frac{1}{n}$ where $m>n\ge 1$, then

$$ \eth ({\mathscr {L}}\zeta ,{\mathscr {L}}\varpi )=\eth (\frac{1}{12m},\frac{1}{12n}), {\mathcal {C}}(\zeta ,\varpi )= {\mathcal {D}}(\zeta ,\varpi )~\ge \frac{1}{n}-\frac{1}{m}~ \text {or}~ {\mathcal {C}}(\zeta ,\varpi )= {\mathcal {D}}(\zeta ,\varpi )=6. $$

Thus,

$${\hat{\phi }}\left( \frac{12}{5}\eth ({\mathscr {L}}\zeta ,{\mathscr {L}}\varpi )\right) \le \frac{{\mathcal {C}}(\zeta ,\varpi )}{5} ={\hat{\phi }}({\mathcal {C}}(\zeta ,\varpi ))-{\hat{\psi }}({\mathcal {D}}(\zeta ,\varpi )). $$

Hence, we have the conclusion from Theorem 2.1 as all assumptions are fulfilled. $\square$

### *Example 2.16*

Let $d:{\mathfrak {P}}\times {\mathfrak {P}} \rightarrow {\mathfrak {P}}$ be a metric with ${\mathfrak {P}}=\{\mho /\mho :[a_1,a_2] \rightarrow [a_1,a_2]~ \text {continuous}\}$ and

$$\eth (\mho _1,\mho _2)=\sup _{\varepsilon \in [a_1,a_2]}\{|\mho _1(\varepsilon )-\mho _2(\varepsilon )|^2\}$$

for every $\mho _1,\mho _2 \in {\mathfrak {P}}$, $0 \le a_1<a_2$ with $\mho _1 \preceq \mho _2$ implies $a_1\le \mho _1(\varepsilon ) \le \mho _2 (\varepsilon )\le a_2, \varepsilon \in [a_1,a_2]$. A self mapping ${\mathscr {L}}$ on ${\mathfrak {P}}$ defined by ${\mathscr {L}} \mho = \frac{\mho }{5}, \mho \in {\mathfrak {P}}$ has a unique fixed point with ${\hat{\phi }}(\ddot{a})=\ddot{a}$ and ${\hat{\psi }}(\ddot{a})=\frac{\ddot{a}}{3}$, for any $\ddot{a} \in [0, +\infty ]$.

### *Proof*

Since, $\min (\mho _1,\mho _2) (\varepsilon )=\min \{\mho _1(\varepsilon ),\mho _2(\varepsilon )\}$ is continuous, and all other assumptions of Theorem 2.3 are satisfied for ${\mathcalligra {s}}=2$. As a result, $0 \in {\mathfrak {P}}$ is the only fixed point of ${\mathscr {L}}$.

$$\square $$

## Limitations

In complete partially ordered *b*-metric space, the existence and uniqueness of a fixed point for a self mapping which satisfies a generalized weak contraction condition with two rational auxiliary functions are discussed. These results are further generalized for two self mappings in the same context and proved the existence of coincidence point, coupled coincidence point and coupled common fixed points. Also, shown that these results are generalized the well known existing results in the literature. Some numerical examples are given to justify the obtained results.

- These results can be extended by involving more mappings in partially ordered *b*-metric space to acquire triple, quadruple fixed points.
- These contractions can be used to obtain a coincidence point, coupled coincidence point and coupled common fixed points for the mappings in various ordered metric spaces with required topological properties like monotone non-decreasing, mixed monotone, compatible etc.

## Data Availability

Not applicable.
